# Botulinum Toxin A Treatment in HIV Infected Patients—A Long-Term Observational Study

**DOI:** 10.3390/jcm11082197

**Published:** 2022-04-14

**Authors:** Stefan Evers, Alexandra Buchheister, Doris Reichelt, Ingo W. Husstedt, Achim Frese

**Affiliations:** 1 Faculty of Medicine, University of Münster, 48149 Münster, Germany; 2 Department of Neurology, Lindenbrunn Hospital, 31863 Coppenbrügge, Germany; 3 Department of Neurology, University of Münster, 48149 Münster, Germany; buchhea@ukmuenster.de (A.B.); hussedt@ukmuenster.de (I.W.H.); 4 Department of Internal Medicine D, University of Münster, 48149 Münster, Germany; reichelt@uni-muenster.de; 5 Academy for Manual Medicine, 48149 Münster, Germany; fresea@uni-muenster.de

**Keywords:** HIV infection, botulinum toxin, dystonia, spasticity

## Abstract

Objective: It is unknown whether interactions between HIV infection and the safety of botulinum toxin A (BTX) exist. Methods: We studied eight patients with HIV infection who were treated with BTX every three months for up to nine years. All patients were on antiretroviral treatment. The efficacy and safety of BTX were evaluated. Results: Indications for BTX treatment (including off-label use), dosage of BTX, and frequency of application did not differ as compared to non-HIV infected patients. BTX treatment was effective in all HIV infected patients during a long-term observation period without loss of efficacy and without clinically relevant side effects. Only one of the eight patients showed mild side effects due to BTX, and no clinical signs of antibody development were noted. We also observed no signs of interaction with antiretroviral treatment. CD4+ cell count and viral load remained stable during the observation period. Conclusions: We conclude that BTX treatment is safe and effective in the treatment of HIV infected patients who suffer also from a condition which can be treated by BTX. It is a therapeutic option in addition to oral medication for HIV infected patients.

## 1. Introduction

Botulinum toxin A (BTX) is a therapeutic option showing good efficacy in the treatment of different neurological symptoms. Main indications for BTX treatment comprise focal dystonia, spasticity, and—only for onabotulinum toxin B—chronic migraine [1,2]. BTX specifically acts on the cholinergic nerve terminals and inhibits the exocytosis of acetylcholine. The consequence is a significant, reversible reduction of neuromuscular transmission. Mechanisms in other indications such as chronic migraine are not yet clarified. In the last decades, BTX has turned to be a safe and well established treatment in neurology. In contrast to other accompanying disorders or symptoms, almost no data are available with regard to the application of BTX in human immunodeficiency virus (HIV) infected patients. The fear for immunological complications due to HIV infection and for pharmacological interactions due to combination antiretroviral therapy (cART) might explain this observation.

Clinically relevant movement disorders are identified in about 3% of all patients suffering from HIV infection [3]. These include tremor, hemiballism, hemichorea, dystonia, myoclonus, tics, and dyskinesia. Movement disorders during HIV infection most often occur just by coincidence. However, they can be caused by opportunistic infections damaging basal ganglia. In addition, it is probable, but not proven, that the HIV itself can cause movement disorders by affecting dopaminergic neurotransmission in the CNS. In particular, the basal ganglia are susceptible to the neurotropism of the HIV [4,5]. Furthermore, spasticity due to opportunistic CNS infection or to the primary HIV manifestation in the CNS can be a reason to consider BTX treatment in HIV infected patients. Also, off-label use of BTX can be justified in, for example, pain syndromes.

As hospitals with both a specialized BTX clinic and a clinic for neurological manifestations of HIV infection, we were able to observe the treatment of HIV infected patients with BTX for long periods. In this paper, we report on the long-term safety and tolerability of BTX in HIV infected patients including data on the efficacy in these patients.

## 2. Methods

We performed a retrospective chart analysis of all HIV infected patients of the Department of Neurology, University of Münster, and of the Department of Neurology, Krankenhaus Lindenbrunn, and selected those who were treated with BTX between 1996 and 2018 at least once. We registered demographic, clinical, laboratory, and neurological data and analysed the efficacy, safety, and tolerability of BTX treatment. The administration of BTX was performed according to the individual judgement of a physician experienced and board certified in BTX treatment (SE, AB). The dosage, sites, and timeline of BTX administration followed standard recommendations as provided in the scientific literature. The efficacy of BTX was judged by clinical examination and, in addition, if needed, by the extensor digitorum brevis test [6].

## 3. Results

### 3.1. Patient 1

A 42-year-old man complained about increased muscle tone in the right hand and forearm leading to problems of coordination about seven years after the diagnosis of HIV infection was made. He received cART including tenofovir, emtricitabine, and atazanavir. Laboratory analysis before BTX treatment showed 670 CD4+ cells/µL, and plasma viral load was <50 copies/mL. The HIV stage was CDC B2; as a neuromanifestation of the HIV infection, the patient suffered from a mononeuropathy of the right sciatic nerve independently from the dystonia. Family history, MRI of the brain including the cervical spine, and analysis of cerebrospinal fluid (CSF) were unremarkable. There was no history of neuroleptics. All diagnostic work-up did not reveal any underlying cause for this movement disorder. Therefore, idiopathic segmental dystonia was diagnosed. However, the induction of dystonia by the antiretroviral treatment cannot be excluded completely. Treatment with L-dopa, trihexyphenidyl, tetrabenazine, and baclofen was not successful over two years, and treatment with BTX was initiated. The patient received 200 mouse units (MU) of BTX in the flexor carpi radialis muscle and in the flexor carpi ulnaris muscle and did not exhibit any side effects. There was profound and constant alleviation of the segmental dystonia during an observation period of more than five years. However, fluctuations of the dystonia during the day course remained. CD4+ cell count (820/µL) and viral load (<50 copies/mL) remained stable during the observation period of nine years.

### 3.2. Patient 2

A 39-year-old male patient had been diagnosed with HIV and infected with stage CDC C3 with a CD4+ cell count of 407/µL and a plasma viral load of 45,000 copies/mL. cART was started, consisting of nevirapine, zidovudine, and lamivudine. Five years later, the patient developed spastic tetraparesis that was more pronounced on the right side, in addition to gait disturbances. Dissociated sensory loss below Th10 dermatome was detected accompanied by urinary incontinence. MRI of the brain was unsuspicious, and MRI of the spinal cord showed increased signal hyperintensity from Th8 to Th10. Analysis of CSF resulted in normal cell count and elevated protein; viral load in CSF was <50 copies/mL. The diagnosis of HIV associated vacuolar myelopathy was made. Treatment with baclofen in a dose of 75 mg/d was without improvement over six months. Treatment with BTX (total dose 250 MU) in the left ischiocrural muscles and in the adductor muscles, in the gastrocnemius muscle, in the flexor digitorum longus muscle, in the soleus muscle, in the tibialis posterior muscle, and in the flexor digitorum profundus muscle on both sides was started. Injections were continued every three months for up to six years. At the beginning of the treatment, the modified Ashworth scale (MAS) score was 4. During the last two years, dosage was augmented to 500 MU. BTX achieved good results in alleviating spasticity without any side effects, and the MAS score was 2. No interactions with cART could be detected. cART consisted of nevirapine, emitricitabine, and tenofovir. The CD4+ cell count was stable at 655/µL, with a recent plasma viral load of <50 copies/mL.

### 3.3. Patient 3

A 54-year-old male patient was referred with cognitive disturbances and finally diagnosed as an HIV infected late presenter in CDC stage C3. The CD4+ cell count was 56/µL, and the plasma viral load was 45,660 copies/mL. A cART, including raltegravir, stavudine, and nevirapine was started, inducing good immunological recovery. Three years later, the patient decided to stop cART, and nine months later, left sided hemiparesis developed. The CD4+ cell count was 37/µL and the plasma viral load was 34,760 copies/mL. Progressive multifocal leukencephalopathy (PML) of the brain was diagnosed by CSF analysis detecting JC virus and typical lesions in MRI (in particular cerebellar lesions). cART including lamivudine, tenofovir, and efavirenz was started, which stopped progression of PML. Two years later, progressive cerebellar tremor developed with severe problems during all activities of daily life. Treatment using primidone and quetiapine was without any effect. Treatment with BTX of the right biceps brachii muscle, brachioradialis muscle, flexor carpi radialis muscle, flexor carpi ulnaris muscle and tibialis posterior muscle in a total dosage of 130 MU was started. The therapy induced a good reduction of cerebellar tremor in amplitude and improved activities of daily living enormously. This therapy continued every three months over a period of four years. The side-effects of BTX did not emerge. No interactions with cART could be detected. cART consisted of stavudine, raltegravir, and lopinavir. The CD4+ cell count was stable, showing at least 345/µL, and the plasma viral load was <37 copies/mL.

### 3.4. Patient 4

A 43-year-old man was hospitalised because of slowly progressive left-sided spastic hemiparesis. A diagnosis of HIV infection stage CDC C3 with a CD4+ cell count of 82/µL and a plasma viral load of 37,560 copies/mL was made. PML was diagnosed by CSF analysis detecting JC virus and typical MRI lesions. A cART including lopinavir, nevirapine, and zidovudine was started. In addition, cidofovir for treatment of PML was given. Treatment of spasticity and severe pain by baclofen was initiated. Already at a low dosage of 10 mg/d, severe side effects such as fatigue, dizziness, and headache occurred. Therefore, treatment of the upper extremity using BTX was started. A total dosage of 60 MU BTX in the left trapezius muscle and biceps muscle was applied. In the follow-up-examinations, a significant reduction of spasticity (from MAS score 3 to MAS score 1) and pain (from 7 on the numeric rating scale to 2) was observed. BTX therapy has now been occurring every three months over five years. There were no side-effects of BTX. No interactions with cART could be detected. cART consists now of tenofovir, emtricitabine, and lopinavir. The CD4+ cell count is stable, showing at least 675 CD4+ cells/µL, and the plasma viral load was <37 copies/mL.

### 3.5. Patient 5

A 52-year-old female patient had been diagnosed with HIV infection for about 10 years in CDC stage C2 when she presented with pain in the neck and shoulders. Physiotherapy and treatment with NSAIDs gave her only partial relief, and NSAIDs were poorly tolerated. The CD4+ cell count was 782/µL, and the plasma viral load was <37 copies/mL. Beside typical distal symmetric polyneuropathy both clinically and in neurophysiological testings, no other manifestations of HIV infection could be detected at the time of presenting. The patient felt healthy otherwise. She was on cART, including tenofovir, stavudine, and emtricitabine. Since the patient was highly impaired by the pain, treatment with BTX was started. The injection was made according the follow-the-pain concept, with 25 MU in the splenius capitis muscle and in the trapezius muscle on either side (total dose 100 MU). BTX treatment decreased the average pain significantly after about two weeks from 7 to 3 on a numeric rating scale. After the second injection, the patient complained of weakness of the neck with mild dropping of the head for some weeks. The exact injection sites were re-evaluated but no change of total dosing was made due to the wishes of the patient. The observation period is ongoing with recurrent very mild head dropping as a side effect of BTX. No interactions with cART have been detected. CD4+ cell count is stable, showing 775 CD4+ cells/µL and a plasma viral load of <37 copies/mL.

### 3.6. Patient 6

A 58-year-old female patient had been diagnosed with HIV infection about 10 years previously and was in CDC stage C3 when she presented with a spastic tretraparesis. This had started about two years ago and lead to the detection of PML. After cART was introduced, PML activity stopped and the spasticity was not progressive any longer. She had spasticity in her distal right arm and distal left leg. Physiotherapy gave her only partial relief, and tizanidine and lioresal were not tolerated. The CD4+ cell count at time of admission was 902/µL, and the plasma viral load was <50 copies/mL. The patient complained of cognitive slowing but felt healthy otherwise. She was on cART. Since the patient was highly impaired by the spasticity (MAS score 4), treatment with BTX was started in the flexor muscles of the right arm (200 MU) and the gastrocnemius and tibialis posterior muscle of the left leg (50 MU each). After the first injection, the patient reported improvement of spasticity but was not yet satisfied with the result (MAS score 3). We increased the dose to 300 MU in the arm and added the soleus muscle of the left leg with 50 MU, resulting in a total dose of 450 MU. We have been injecting this patient now for over four years and could not detect any interactions with cART. The MAS scores for both arm and leg were consistently 2.

### 3.7. Patient 7

A 48-year-old male patient with HIV infection for over 12 years due to intravenous drug abuse presented with slowly progressive tetraparesis. At the time of hospital admission, he was very weak (BMI 16.7 kg/m^2^) and showed both spasticity including Babinski’s sign and muscular atrophy and fasciculations. The electromyography confirmed positive sharp waves and denervation in all extremities. In addition, we could observe tongue fibrillation. A diagnosis of amyotrophic lateral sclerosis (ALS) was made, possibly induced by HIV infection, which has been described in the literature [7]. He was under cART (bictegravir, emtricitabine, tenofoviralafenamid) but the CDC4+ cell count was low (under 200/µL) and the viral load was regularly above 100,000 copies/mL. One major complaint of the patient was spasticity in the adductor muscles of both legs so that he could not sit or move when lying in bed (MAS score 4). We injected 200 MU on each side to improve mobility of the legs. The patient reported a better control of the legs during a period of four injection intervals (MAS score 3). His situation then deteriorated and he died in hypercapnic coma.

### 3.8. Patient 8

A 49-year-old male patient attended the outpatient clinic because of right-sided facial pain. He developed this pain some weeks after a typical zoster ophthalmicus. The pain was located around the eye and in the distribution of the second trigeminal nerve branch with a mean intensity of 6 out of 10 on a numeric rating scale. The zoster manifestation was the reason to perform a blood test for HIV infection, which was positive. The patient showed at that time a CD4+ cell count of 302/µL with a viral load of 1000 copies/mL but was otherwise healthy and had no other symptoms of HIV infection; he received cART for about six months at admission. The neurological examination was unremarkable. The patient was treated with pregabalin up to 150 mg bid which gave him about 50% relief. He did not tolerate higher doses of pregabalin and refused to take any antidepressants. He wished further pain treatment without oral drugs and was therefore offered BTX which has some evidence in purely trigeminal pain treatment but is off label [8]. Under BTX 25 MU injected subcutaneously in the distribution of the right second trigeminal branch, he experienced a further 30% reduction in pain and was satisfied with the effect. After three cycles of injection, the effect remained stable.

## 4. Discussion

The application of BTX represents a very rare therapy in HIV infected patients although it is very effective for movement disorders, spasticity, and in other off-label indications. Our patient sample is the first case series reporting that BTX can be administered to adult patients with HIV infection easily for approved indications such as spasticity and dystonia (Table 1). In our experience, application of BTX in this patient group is safe. We observed an improvement of gait function or reduction of tremor or spasticity and relief of pain as expected from other patients without HIV infection. We could not observe any interaction between cART and BTX. Furthermore, we could not see any spread of the toxin effect distant to the site of injection. In all patients but one we used BTX for intramuscular injection. This showed no side effects except minor and tolerable weakness in one patient. All patients who were treated with BTX continued this treatment, and there was no non-responder.

We performed a thorough diagnostic work-up including MRI and CSF analysis of every single patient to clarify possible associations between HIV infection and neuromanifestations for which they were treated with BTX. Our patients had developed both primary neuromanifestations of HIV infection (HIV associated neurocognitive disorder; vacuolar myelopathy; polyneuropathy) and secondary neuromanifestations (PML; possibly postherpetic neuralgia). In two of eight cases (#3 and #4), we observed a link between opportunistic infection (PML) and neurological symptoms. In one patient (#2), there was a primary neuromanifestation of HIV infection (vacuolar myelopathy), in one patient (#1) the link remains unclear (focal dystonia), and in one patient (#5) there is probably no relation between HIV infection and indication for BTX treatment. Treatment of spasticity in two patients and of dystonia in one patient was in accordance with the indications BTX is approved for. If spasticity is taken as an indication for BTX in general, we administered BTX outside its approved indications only in three of our eight patients (for cerebellar tremor, myofascial pain, trigeminal pain).

The patients were treated regularly in three month intervals with BTX. The HIV infected patients in our study reported the onset of improvement about six days after treatment onset, the maximum improvement was about six weeks after treatment onset, and a diminishing efficacy after about 10 weeks similar to reports of patients without HIV infection was noticed. Therefore, we assume a similar efficacy profile in both groups.

According to our clinical impression, no neutralizing antibodies to BTX have been developed. Even after up to nine years of application, there was no therapeutic failure. Development of antibodies, although rare, represents an important limitation to the application of BTX in general. It might be that the immunosuppressive properties of cART induce a protection against the development of antibodies and that therefore no development of antibodies was observed.

All patients but two (No. 4 and No. 7) received BTX treatment in a state of good immunocompetence. Viral loads were low or undetectable under cART therapy, CD4+ cell count was normal and kept stable over the time of treatment in six patients. As we have treated only two patients in a severe immunosuppressed state, we cannot be sure about safety and tolerability in immunocomprised patients.

The results of this study are in accordance with reports on the application of BTX in four HIV infected children suffering from spasticity [9] and in a pilot study in five HIV infected adults with cosmetic facial problems [10] who also showed no side-effects and good efficacy over more than two years.

In summary, BTX represents an effective treatment for spasticity and movement disorders in HIV infected patients with a reasonable safety-efficacy profile when conventional treatment (e.g., baclofen) is not tolerated or ineffective. In addition, the off-label use of BTX in pain treatment also showed good results in two patients with HIV infection.

## Figures and Tables

**Table 1:** Data of five patients with HIV infection treated with botulinum toxin A for different indications.

Patient	Age	Sex	Neurological Signs	BTX Type and Dose	CDC Stage	HIV Neuromanifestations	Injected Muscles	Observation Time
1	42	M	segmental dystonia of right arm	200 MU OnaBTX	B2	mononeuropathy of the right sciatic nerve	flexor carpi radialis, flexor carpi ulnaris	nine years
2	39	M	right leg spasticity	250 to 500 MU OnaBTX	C3	HIV vacuolar myelopathy	ischiocrural, gastrocnemius, flexor digitorum	nine years six years
3	54	M	cerebellar tremor	130 MU OnaBTX	C3	HIV polyneuropathy, HAND, PML	flexor carpi radialis, flexor carpi ulnaris, brachioradialis	four years
4	38	M	left-sided spastic hemiparesis	50 to 60 MU IncoBTX	C3	condyloma accuminata, PML	trapezius, biceps brachii	five years
5	52	F	myofascial pain syndrome	100 MU IncoBTX	C2	HIV polyneuropathy	splenius capitis, trapezius	four years
6	58	F	spastic tetraparesis (dominantly left-sided)	450 MU IncoBTX	C3	PML	soleus, gastrocnemius left arm flexor	four years
7	48	M	general spasticity of legs	200 MU IncoBTX	C3	ALS associated with HIV	adductor group bilateral	one years
8	49	M	postherpetic neuralgia	25 MU IncoBTX	B2	zoster ophthalmicus	second trigeminal branch	nine years

CDC = Center for Disese Control; HAND = HIV associated neurocognitive disorder; PML = Progressive multifocal leukencephalopathy; HIV = Human immunodeficiency virus.
